# An open‐source, expert‐designed decision tree application to support accurate diagnosis of myeloid malignancies

**DOI:** 10.1002/jha2.182

**Published:** 2021-03-26

**Authors:** Thomas Coats, Daniel Bean, Theodora Vatopoulou, Dhanapal Vijayavalli, Razan El‐Bashir, Aikaterini Panopoulou, Henry Wood, Manujasri Wimalachandra, Jason Coppell, Patrick Medd, Michelle Furtado, David Tucker, Austin Kulasakeraraj, Joya Pawade, Richard Dobson, Robin Ireland

**Affiliations:** ^1^ Department of Haematology Royal Devon and Exeter NHS Foundation Trust Exeter UK; ^2^ Biostatistics and Health Informatics King's College London London UK; ^3^ Health Data Research UK London University College London London UK; ^4^ Department of Haematology St George's University Hospitals NHS Foundation Trust London UK; ^5^ Department of Haematology Medway NHS Foundation Trust Kent UK; ^6^ Department of Haematological Medicine King's College Hospital NHS Foundation Trust London UK; ^7^ Department of Haematology Darent Valley Hospital Kent UK; ^8^ Department of Haematology Royal Marsden NHS Foundation Trust London UK; ^9^ Department of Haematology Derriford Hospital Plymouth UK; ^10^ Department of Haematology Royal Cornwall NHS Trust Truro UK; ^11^ Department of Pathology North Bristol NHS Trust Bristol UK

**Keywords:** classifications, clinical haematology, diagnostic haematology, myeloid leukaemia

## Abstract

Accurate, reproducible diagnoses can be difficult to make in haemato‐oncology due to multi‐parameter clinical data, complex diagnostic criteria and time‐pressured environments. We have designed a decision tree application (DTA) that reflects WHO diagnostic criteria to support accurate diagnoses of myeloid malignancies. The DTA returned the correct diagnoses in 94% of clinical cases tested. The DTA maintained a high level of accuracy in a second validation using artificially generated clinical cases. Optimisations have been made to the DTA based on the validations, and the revised version is now publicly available for use at http://bit.do/ADAtool.

## 1 INTRODUCTION

National institute for health and care excellence (NICE) guidance recommends multi‐disciplinary meetings (MDMs) as best practice in the diagnostic work‐up of suspected haemato‐oncology patients. Compared to some areas of medicine, haematological malignancies are already a very well‐studied discipline. A sizeable proportion of treatments are based on randomised controlled trial data, and with regard to the diagnostic criteria, the commonly used WHO Classification of Tumours is supported by 4500 references [1, 2].

Despite the wealth of information to support clinicians, making accurate diagnoses in a busy MDM can be difficult—this same abundance of information can be difficult to navigate, is subject to periodic updates and requires the integration of multiple sources of data across different platforms (clinical, morphological, genetic, radiological), all in a time‐pressured environment [3]. Poor documentation may lead on to inefficiencies of clinical service, incorrect treatment strategies and poor quality of data for local and national audit [4].

Technology is widely recognised as offering an opportunity to improve the accuracy and efficiency of diagnostics in medicine [5, 6]. These include big data analyses of hospital records, artificial intelligence or automated algorithms to transform how we care for patients.

The use of algorithms is not new in diagnostic healthcare; they help to standardise how a diagnosis is reached and are widespread in every‐day clinical practice [7]. Typically, these algorithms are described and shared as text documents or flowcharts and are designed for human use, not for automated or computational use [8, 9]. These formats are also not amenable to automated testing or verification. However, many of the diagnostic criteria in the WHO are clearly set out and therefore give the possibility of designing a digitised algorithm to support clinicians with the complexity of the diagnostics.

We have previously shown that digital solutions can improve the accuracy of MDS‐subtyping in a single‐centre retrospective study [10]. We have now developed a comprehensive decision tree application (DTA) that recapitulates the 2016 WHO diagnostic criteria for adult myelodysplastic syndrome (MDS), acute myeloid leukaemia (AML), myeloproliferative neoplasm (MPN) and myelodysplastic/myeloproliferative syndrome (MDS/MPN) disease categories. This multi‐centre study set out to validate the DTA by independent testing. It shows our DTA is accurate and can improve precision in the diagnosis of commonly occurring myeloid malignancies.

## 2 METHODS

The DTA was designed using the open source platform esyN and is publicly available as a web application [11]. The DTA clinical algorithms were derived using the WHO criteria transformed into esyN using a graphical interface which does not require writing code. Users enter clinical data within the following categories: full blood count readings, bone marrow morphology/immunophenotyping, cytogenetics, molecular and clinical information. Clinical data are fed into the decision tree, and a WHO diagnosis is displayed. Thirty‐five different myeloid diagnoses, as defined by WHO criteria, are available within the DTA, and a ‘non diagnostic’ outcome. Acute leukaemias with ambiguous lineage, myeloid neoplasms with germline predisposition, myeloid sarcoma, myeloid proliferations associated with Down syndrome, mastocytosis, blastic plasmacytoid dendritic cell neoplasam and JMML are not included.

A clinician at each centre received training on using the DTA. Cases with a diagnostic bone marrow, documented local MDM discussion and a WHO diagnosis were selected for analysis. Clinical data were logged using the DTA, and the suggested diagnosis was recorded. No clinical data were uploaded to or recorded in esyn.org. All analyses were run locally in a web browser. In cases of discrepancy between the DTA and the MDM outcome, a second independent clinical opinion was sought to identify whether (i) the DTA had suggested an incorrect diagnosis or (ii) the MDM diagnosis needed revision.

A second validation was performed by creating 10,000 in silico case reports with random values for the different clinical variables. Each case that was selected for subsequent validation covered a unique route through the decision tree—this ensured the whole tree was tested, with additional robustness from testing unpredictable combinations of variables. Each case report was reviewed by two haematologists working independently to establish the most appropriate diagnosis for the artificial cases (a team of eight FRCPath‐certified haematologists performed this step). Any case where there was discrepancy between the DTA and either doctor was reviewed by a team of three independent haematologists to adjudicate on the most likely diagnosis. Any of the randomly generated cases that were considered highly improbable by the independent review were removed from further analysis.

Accuracy and F1 scores (the harmonic mean of precision and recall) were calculated using scikit‐learn 0.20.3 in Python 3.7.0.

## 3 RESULTS AND DISCUSSION

**FIGURE 1:**
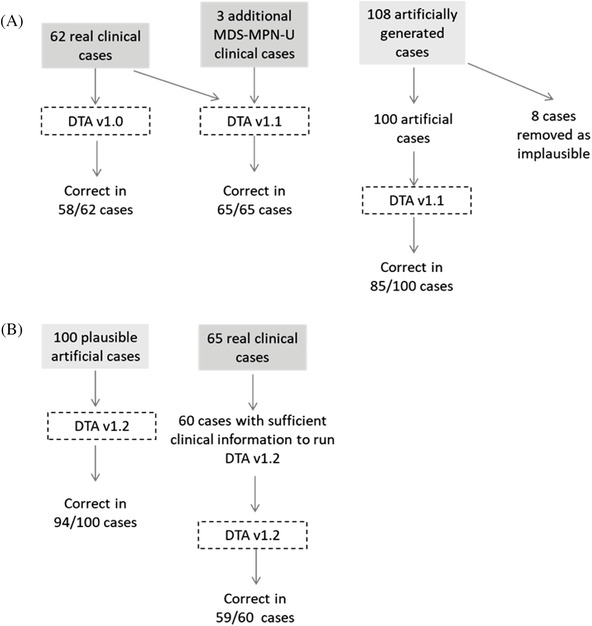
Schema summarising workflow of cases run through the decision tree application (DTA) and the DTA accuracy

Sixty‐two cases of myeloid malignancy were logged in the DTA from five centres, covering 24 of the possible 35 diagnoses in the DTA (Figure 1). Fifty‐five cases (89%) were concordant between the DTA and the MDM diagnosis. On independent review of the seven discordant cases, three cases were recommended to revise the MDM diagnosis to the DTA‐generated diagnosis. Four cases were discordant due to an error in the logic coded in the DTA. Therefore, overall the DTA was accurate in 58 of 62 cases (94%). An interesting observation from one myeloid case was that greater clarity was required from the trephine report to be able to run the DTA. When the biopsy was reviewed and the report updated, this led to a recommended revision of the MDM diagnosis.

Subsequently, minor adjustments were made to improve the DTA accuracy (DTA version 1.1). For example, three of the DTA errors were due to one missing logic step for cases of myelodysplastic/myeloproliferative syndrome, unclassifiable (MDS‐MPN‐U). The clinical data from the 62 cases were re‐run through the updated DTA, and all 62 cases gave an output that matched the amended MDM diagnosis. To further validate the updated DTA, three additional cases with a known diagnosis of MDS‐MPN‐U that would have been mis‐diagnosed by the original version, were tested; the correct diagnosis was returned in all three.

To further test the DTA, 108 randomly generated clinical cases that covered all of the major branches and diagnoses within the DTA were selected for validation by pairs of clinicians working independently. Eight cases were subsequently removed for being implausible. After independent review, the DTA was found to be incorrect in 15 of 100 cases, giving an overall accuracy of the DTA of 85%. The average accuracy for the individual doctors was 53% (range 43%–71%).

**FIGURE 2:**
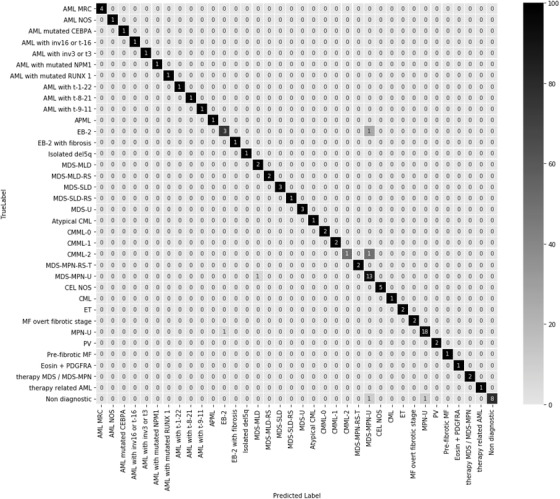
Performance of 100 artificially generated cases run through DTA v1.2

Adjustments to the DTA were made to reflect the feedback from the panel of independent experts (DTA version 1.2) (Figure 1). The 100 plausible randomly generated cases were re‐run through the DTA. Ninety‐four cases had agreement between the diagnosis and the updated DTA outcome giving an overall accuracy of 94% and misclassification rate of 6%. The DTA is most accurate in correctly categorising a case where a myeloid malignancy is confirmed, as opposed to discriminating a non‐diagnostic case from a malignant diagnosis, with high F1 scores (>0.80) and accuracy (>0.90) returned for all WHO categories (Figure 2). Sixty of the original 65 clinical cases had sufficient clinical information to run on the updated DTA. For 59 of the 60 cases, the correct diagnosis was returned. These two validations show that an algorithmic approach is amenable to sequential optimisations leading to high levels of accuracy.

The revised version of the DTA is available for use at http://bit.do/ADAtool.

The accuracy of the DTA was lower when processing randomly generated cases compared to genuine clinical cases. The case selection was designed to test the decision boundaries between diagnoses; so many cases only had small differences between them, or nearly fit the criteria for an alternative diagnosis. Feedback from clinicians assigning diagnoses to these cases was that there was more heterogeneity of clinical features than normally found in routine clinical practice. These factors may explain the lower than expected accuracy of the individual doctors. The ability of the DTA to perform well in this context suggests that the algorithm is durable even with artificially generated, atypical presentations.

The review process of the artificially generated cases demonstrated some clinical scenarios where consensus was difficult to reach. For example, establishing clear criteria for MPN‐U was difficult. Applying an algorithmic approach also highlights the areas in the WHO diagnostic criteria that are more open to interpretation, for example, whether the presence of a non‐reactive thrombocytosis would re‐classify an otherwise typical chronic myelomonocytic leukaemia (CMML) or chronic eosinophilic leukaemia, not otherwise specified (CEL‐NOS), case into an MDS‐MPN‐U. In these circumstances, a differential diagnosis of valid WHO diagnoses is likely to be more appropriate, and future iterations of the DTA will look to address this.

## 4 CONCLUSION

These DTA provide an example of how technology can be used to support clinicians’ assessment of multi‐parameter, complex data, to produce improved diagnostic accuracy and facilitate MDM recording of outcomes. The DTA can highlight additional tests that are required to advance the diagnostic pathway or indicate relevant data points that are missing. Its use encourages a consistent reporting approach and could also be useful in education. As illustrated in this study, adjustments can be made to the DTA and therefore incorporate new diagnostic criteria that can be applied to both recent and historical patients for audit, research or direct clinical care. Further work is now required to expand the validation to a larger cohort of real clinical cases, establishing safety for use in clinical practice.

DTA was sequentially optimised from version v1.0 to v1.1 and v1.2. (**A**) DTA v1.0 was tested on cohort of 62 real clinical cases. DTA v1.1 was run on original 62 cases plus three additional MDS‐MPN‐U cases. One hundred and eight artificially generated cases were created and run through DTA v1.1. (**B)** DTA v1.2 was re‐run on 100 artificially generated cases and on 60 of the original 65 clinical cases (five clinical cases lacked sufficient information to run cases through DTA v1.2).

Confusion matrix demonstrating the performance of the whole DTA tree assessed by DTA v1.2 analysing 100 artificially generated cases. A correct diagnosis is where the WHO diagnosis returned by the DTA (Predicted label) matches the actual diagnosis (True label). The numbers show the percentage of the 100 cases with corresponding predicted‐true label combinations. Matching prediction and true labels are found along the diagonal. Colour shading represents the percentage recall (0%–100%) for each diagnosis tested.

## Supporting information

Supporting Information

## Data Availability

Source results from patient records used at all sites in the study will not be available due to inability to safely fully anonymise up to the Information Commissioner Office (ICO) standards given the highly sensitive and granular nature of the data (e.g. blood results, genetic tests, diagnoses etc.). A subset of the data that support the findings of this study is available on reasonable request to the corresponding author. The authors declare that there is no conflict of interest that could be perceived as prejudicing the impartiality of the research reported. Research design: Thomas Coats, Daniel Bean, Richard Dobson and Robin Ireland. Collected patient data: Theodora Vatopoulou, Jason Coppell, Aikaterini Panopoulou, Manujasri Wimalachandra and Razan El‐Bashir. Assessment of artificially generated cases: Dhanapal Vijayavalli, Aikaterini Panopoulou, Jason Coppell, Henry Wood, Patrick Medd, Michelle Furtado, David Tucker and Austin Kulasakeraraj. Reviewed the discrepant cases: Theodora Vatopoulou, Joya Pawade and Robin Ireland. Statistical analysis: Thomas Coats and Daniel Bean. Analysis and interpretation of data: Thomas Coats, Daniel Bean, Richard Dobson and Robin Ireland. Drafting of the manuscript: Thomas Coats, Daniel Bean, Richard Dobson and Robin Ireland.
