# Supplementary material for: An open‐source, expert‐designed decision tree application to support accurate diagnosis of myeloid malignancies
Source: EJHaem. 2021 Mar 26;2(2):261–5. doi: 10.1002/jha2.182 (PMC9175663; doi:10.1002/jha2.182)
Supplement: Supplementary file 1 — Supporting Information [file JHA2-2-261-s001.docx]

## Supplementary Material

S1 Supplementary Methods

S2 List of available WHO diagnoses in DTA

S3 Example case report of *in silico* generated clinical case

S4 List of 62 clinical cases tested using DTA v1.0

S5 Performance of 100 artificially generated cases run through DTA v1.1 (prior to optimisation)

S6 Performance of 100 artificially generated cases assessed by individual clinicians

## S1 Supplementary Methods

### Decision Tree design

The DTA was designed by practicing haematologists after careful review of the WHO diagnostic criteria. A hierarchy of disease categories was identified based on these criteria to build the basic structure of the decision tree (e.g. a diagnosis of chronic myeloid leukaemia supersedes a diagnosis of primary myelofibrosis). Additional branches were added to the tree to ensure that the necessary criteria were fulfilled to reach any final diagnosis. Terminal nodes on the decision tree represent a final diagnosis or concluding statement. The overall design was iteratively updated based on internal testing and two rounds of external validation. For any decision tree in esyN the full tree can be viewed in “build” mode and a simplified user interface that does not show the tree itself is available in “tool” mode.

### Decision Tree application instructions

The decision tree application can be found at <http://bit.do/ADAtool>.

Users should note that diagnoses are suggestions only and should not replace careful clinical review, clinician judgement and reference to the WHO guidelines.

To run the application, enter the relevant clinical information in the boxes and select the ‘RUN’ button. All boxes marked with an asterisk must have some information added in order to run the DTA. You will be prompted if further information is required to establish a diagnosis.

It is left to clinician discretion as to how certain variables are arrived at. For example, blast % can be drawn from aspirate morphology, trephine immunohistochemistry, immunophenotyping or a combination of all three. As in routine clinical practice the ultimate decision as to which test is given prominence is left with the clinician.

Additional written instructions are available here:

<https://drive.google.com/file/d/1eSLJ0O9jFaBQaW64SDEIe5vmZBuRxHCB/view?usp=sharing>

A video demonstration is available here:

<https://www.youtube.com/watch?v=DaoFvesu6PM>

## S2 List of available WHO diagnoses in DTA

| **WHO Chapter** | **WHO classification of tumours** | **Disease subtypes available in DTA** | **Disease subtypes NOT available in DTA** |
| --- | --- | --- | --- |
| 2 | Myeloproliferative neoplasms | PV, ET, CML, primary overt MF, CNL, MPN-U, pre-fibrotic MF, CEL NOS |  |
| 4 | Myeloid/lymphoid neoplasms with eosinophilia and gene rearrangement | Myeloid/lymphoid neoplasms with *PDGFRA* rearrangement, *PDGFRB* rearrangement, *FGFR1* rearrangement, | *PCM1-JAK2 rearrangement* |
| 5 | Myelodysplastic/myeloproliferative neoplasms | CMML-0, CMML-1, CMML-2, atypical CML, MDS-MPN-RARS-T, MDS-MPN-U | JMML |
| 6 | Myelodysplastic syndromes | MDS-MLD, MDS-SLD, isolated del 5q, MDS-SLD-RS, MDS-EB1, MDS-EB2, MDS-MLD-RS, MDS-U | *Refractory cytopenia of childhood* |
| 8 | Acute myeloid leukaemia and related precursor neoplasms; recurrent mutations | t(8;21), t(16)/inv(16), t(15;17), NPM1, bialleleic CEBPA, t(9;11), t(1;22), t(3)/inv(3), *RUNX1* | *AML with BCR-ABL 1* |
| 8 | Acute myeloid leukaemia and related precursor neoplasms;  AML, NOS | AML NOS | AML with M1-7, acute basophilic leukaemia, acute panmyelosis with fibrosis |
| 8 | Acute myeloid leukaemia and related precursor neoplasms;  AML with myelodysplasia related changes | AML with MRC |  |
| 8 | Therapy related myeloid neoplasm | Therapy related AML, therapy related MDS or MDS-MPN |  |
| - | Other | Non diagnostic |  |

Provisional WHO categories are in italics

Other notable exceptions include:

- acute leukaemia of ambiguous lineage
- myeloid proliferations associated with Down syndrome
- mast cell related malignancies
- myeloid sarcoma
- blastic plasmacytoid dendritic cell neoplasm
- myeloid neoplasms with germline predisposition

## S3 Example case report of *in silico* generated clinical case

Female, a new referral.

**FBC:** Hb 130x10^9/L WBC 2.5x10^9/L Plt 700x10^9/L Neut 1.8x10^9/L Mono 0.075x10^9/L Eos 0.1x10^9/L Baso 0.1x10^9/L

**Blood film:** PB blasts 2%, Promyelocytes, Myeloctyes and Metamyelocytes comprise 0%.

**Bone Marrow:** Hypercellular sample. Normal megakaryocyte numbers. Normal maturation of granulopoiesis. 3 lineages of dysplasia. Dyserythropoiesis present. Dysmegakaryopoiesis present. Dysgranulopoiesis present. No megakaryocyte atypia present. BM blasts 10%. Auer rods present. Fibrosis grade 1. 6% ring sideroblasts seen.

**Cytogenetics:** 2 abnormalities including: del(13q).

**Mutations:** Absent mutations.

**Other:** EPO level normal. LDH high. Splenomegaly present. No reactive cause present, no reactive cause for fibrosis present, No secondary cause for anaemia present eg renal impairment, bleeding etc.

## S4 List of 62 clinical cases tested using DTA v1.0

| **ID** | **DTA v1.0 Diagnosis** | **Actual diagnosis** | **MDT diagnosis (if different from actual diagnosis)** | **DTA v1.0 incorrect?** |
| --- | --- | --- | --- | --- |
| SG1 | MDS-MPN-U | MDS-MPN-U |  |  |
| SG2 | MPN-U | MPN-U |  |  |
| SG3 | PV | PV |  |  |
| SG4 | AML with NPM1 | AML with NPM1 |  |  |
| SG5 | AML with bialleleic CEBPA | AML with bialleleic CEBPA |  |  |
| SG6 | CML | CML |  |  |
| SG7 | MPN-U | MPN-U | ET |  |
| SG8 | Overt MF | Overt MF |  |  |
| SG9 | CMML-0 | MDS-MPN-U |  | Yes |
| MD1 | AML with MDS related changes | AML with MDS related changes |  |  |
| MD2 | AML with MDS related changes | AML with MDS related changes |  |  |
| MD3 | EB-1 | EB-1 |  |  |
| MD4 | CMML-0 | MDS-MPN-U |  | Yes |
| MD5 | AML with 8_21 | AML with 8_21 |  |  |
| MD6 | AML with inv16 or t_16 | AML with inv16 or t_16 |  |  |
| MD7 | AML NOS | AML_MRC |  | Yes |
| MD8 | APML | APML |  |  |
| DP1 | PV | PV |  |  |
| DP2 | CML | CML |  |  |
| DP3 | AML with MDS related changes | AML with MDS related changes |  |  |
| DP4 | CMML-1 | CMML-1 |  |  |
| DP5 | AML with inv16 or t_16 | AML with inv16 or t_16 |  |  |
| DP6 | Overt MF | Overt MF |  |  |
| DP7 | EB-2 | EB-2 |  |  |
| DP8 | MDS_SLD_RS | MDS_SLD_RS |  |  |
| DP9 | AML_NOS | AML_NOS |  |  |
| DP10 | AML with NPM1 | AML with NPM1 |  |  |
| DP11 | Chronic Neutrophilic Leukaemia | Chronic Neutrophilic Leukaemia |  |  |
| DP12 | PV | PV |  |  |
| DP13 | EB-1 | EB-1 |  |  |
| DP14 | t-AML | t-AML |  |  |
| RDE7 | CMML-2 | CMML-2 |  |  |
| RDE8 | Overt MF | Overt MF |  |  |
| RDE10 | therapy related MDS or MDS_MPN | therapy related MDS or MDS_MPN |  |  |
| RDE1 | AML with NPM1 | AML with NPM1 |  |  |
| RDE2 | CML | CML |  |  |
| RDE3 | AML with MDS related changes | AML with MDS related changes |  |  |
| RDE11 | MDS_MLD | MDS_MLD |  |  |
| RDE12 | MDS_SLD | MDS_SLD |  |  |
| RDE13 | CEL NOS | MDS-MPN-U |  | Yes |
| RDE14 | AML with MDS related changes | AML with MDS related changes |  |  |
| RDE15 | AML with NPM1 | AML with NPM1 |  |  |
| KCH3 | CMML-1 | CMML-1 |  |  |
| KCH4 | AML with MDS related changes | AML with MDS related changes |  |  |
| KCH5 | AML with MDS related changes | AML with MDS related changes |  |  |
| KCH6 | therapy related MDS or MDS_MPN | therapy related MDS or MDS_MPN |  |  |
| KCH7 | EB-1 | EB-1 | MDS-MPN |  |
| KCH8 | MDS_MLD | MDS_MLD |  |  |
| KCH9 | Isolated del5q | Isolated del5q |  |  |
| KCH10 | AML with inv16 or t_16 | AML with inv16 or t_16 |  |  |
| KCH11 | APML | APML |  |  |
| KCH12 | CML | CML |  |  |
| KCH13 | therapy related MDS or MDS_MPN | therapy related MDS or MDS_MPN |  |  |
| KCH14 | MDS_MLD | MDS_MLD |  |  |
| KCH15 | Essential Thrombocytosis | Essential Thrombocytosis |  |  |
| KCH16 | t-AML | t-AML |  |  |
| RDE4 | AML with MDS related changes | AML with MDS related changes |  |  |
| RDE6 | Overt MF | Overt MF |  |  |
| RDE9 | therapy related MDS or MDS_MPN | therapy related MDS or MDS_MPN | AML NOS |  |
| RDE5 | EB-2 | EB-2 |  |  |
| RDE18 | AML with MDS related changes | AML with MDS related changes |  |  |
| RDE17 | MDS_SLD | MDS_SLD |  |  |

## S5 Performance of 100 artificially generated cases run through DTA v1.1 (prior to optimisation)


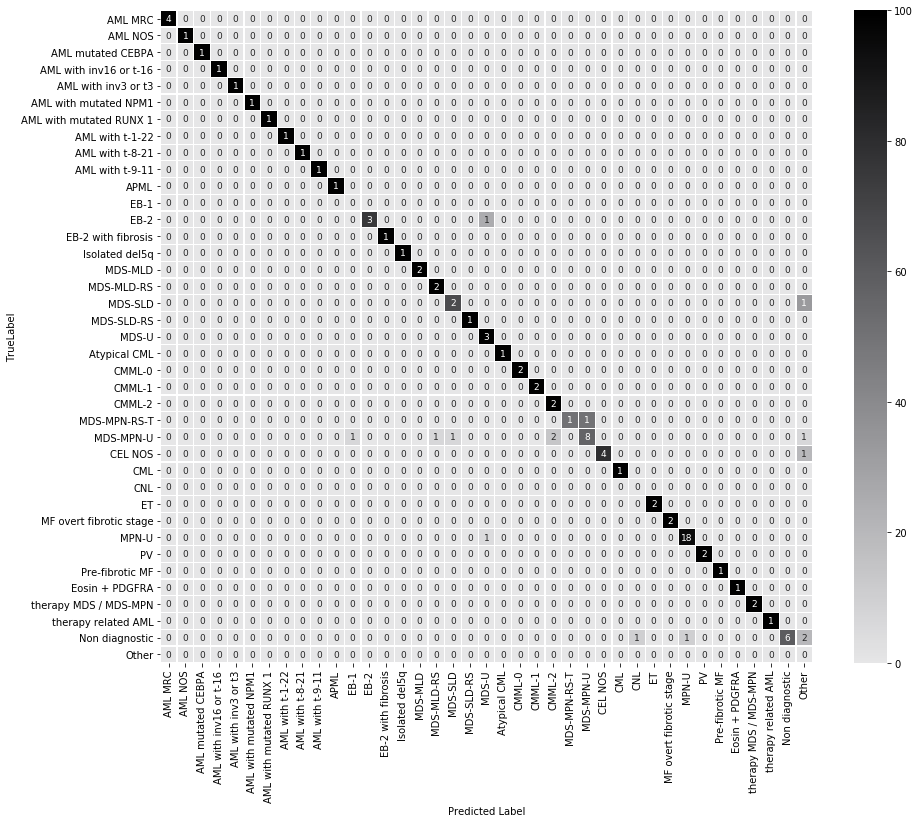


Confusion matrix demonstrating the performance of the whole DTA tree assessed by DTA v1.1 analysing 100 artificially generated cases. A correct diagnosis is where the WHO diagnosis returned by the DTA (Predicted label) matches the actual diagnosis (True label). The numbers show the percentage of the 100 cases with corresponding predicted-true label combinations. Matching prediction and true labels are found along the diagonal. Colour shading represents the percentage recall (0-100%) for each diagnosis tested.

## S6 Performance of 100 artificially generated cases assessed by individual clinicians

##
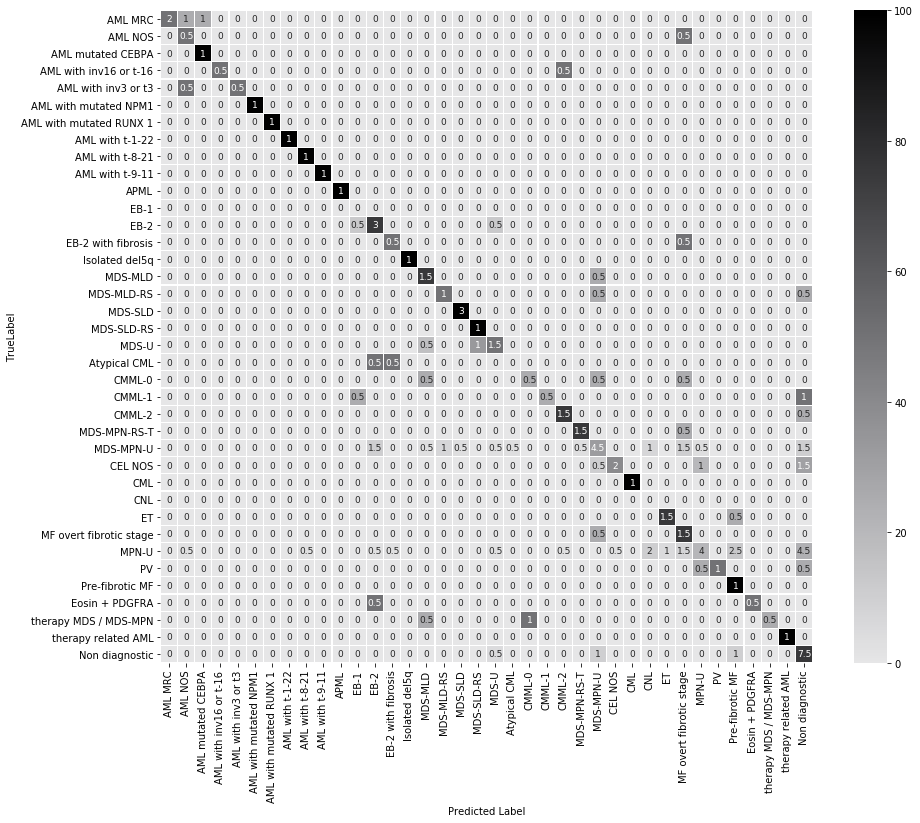


Confusion matrix demonstrating the performance of the whole DTA tree assessed by individual clinicians analysing 100 artificially generated cases. A correct diagnosis is where the WHO diagnosis returned by the clinician (Predicted label) matches the actual diagnosis (True label). The numbers show the percentage of the 100 cases with corresponding predicted-true label combinations. Matching prediction and true labels are found along the diagonal. Colour shading represents the percentage recall (0-100%) for each diagnosis tested.
